# Statistical Properties of Musical Creativity: Roles of Hierarchy and Uncertainty in Statistical Learning

**DOI:** 10.3389/fnins.2021.640412

**Published:** 2021-04-20

**Authors:** Tatsuya Daikoku, Geraint A. Wiggins, Yukie Nagai

**Affiliations:** ^1^International Research Center for Neurointelligence (WPI-IRCN), The University of Tokyo, Tokyo, Japan; ^2^AI Lab, Vrije Universiteit Brussel, Brussels, Belgium; ^3^School of Electronic Engineering and Computer Science, Queen Mary University of London, London, United Kingdom; ^4^Institute for AI and Beyond, The University of Tokyo, Tokyo, Japan

**Keywords:** statistical learning, prediction, creativity, development, hierarchy, abstraction, integration, autism spectrum disorder

## Abstract

Creativity is part of human nature and is commonly understood as a phenomenon whereby something original and worthwhile is formed. Owing to this ability, humans can produce innovative information that often facilitates growth in our society. Creativity also contributes to esthetic and artistic productions, such as music and art. However, the mechanism by which creativity emerges in the brain remains debatable. Recently, a growing body of evidence has suggested that statistical learning contributes to creativity. Statistical learning is an innate and implicit function of the human brain and is considered essential for brain development. Through statistical learning, humans can produce and comprehend structured information, such as music. It is thought that creativity is linked to acquired knowledge, but so-called “eureka” moments often occur unexpectedly under subconscious conditions, without the intention to use the acquired knowledge. Given that a creative moment is intrinsically implicit, we postulate that some types of creativity can be linked to implicit statistical knowledge in the brain. This article reviews neural and computational studies on how creativity emerges within the framework of statistical learning in the brain (i.e., statistical creativity). Here, we propose a hierarchical model of statistical learning: statistically chunking into a unit (hereafter and shallow statistical learning) and combining several units (hereafter and deep statistical learning). We suggest that deep statistical learning contributes dominantly to statistical creativity in music. Furthermore, the temporal dynamics of perceptual uncertainty can be another potential causal factor in statistical creativity. Considering that statistical learning is fundamental to brain development, we also discuss how typical versus atypical brain development modulates hierarchical statistical learning and statistical creativity. We believe that this review will shed light on the key roles of statistical learning in musical creativity and facilitate further investigation of how creativity emerges in the brain.

## Introduction

Creativity is a process of producing something that is both original and worthwhile (Lubart and Mouchiroud, 2003; Kozbelt et al., 2010; Robert, 2011). It also contributes to the perception and production of information in new ways (Dailey et al., 1997; Furlong, 2009; Hargreaves, 2012). Creativity sometimes triggers innovation in science, technology, and arts, creating historical shifts in human society. Over a long period, many people have been fascinated by the question of how creativity emerges in the brain. There is no doubt that creativity is intricately linked to acquired knowledge; however, the underlying mechanisms remain unclear. In particular, there is little understanding of how novel and uncertain information emerges from acquired knowledge and why such uncertain information can be accepted as creative. Recently, a growing body of literature has suggested that *statistical learning* and the knowledge that results therefrom may underlie creativity (Wiggins, 2012, 2020; Daikoku, 2019a,b; Zioga et al., 2019).

Statistical learning is an implicit and innate function of the human brain and is essential for brain development (Saffran et al., 1996). The statistical learning system allows us to “predict” an upcoming phenomenon to minimize prediction error and resolve “perceptual uncertainty” (Friston, 2010; Clark, 2013; Hasson, 2017). More specifically, statistical learning involves a mechanism by which the brain calculates the transitional probability (i.e., local statistics) and uncertainty of its probability distribution (i.e., global statistics). Statistical learning ultimately allows the brain to optimize prior predictions and suppress uncertainty. Through statistical learning, humans acquire the ability to produce and comprehend structured sequences, such as music and language.

Evidence suggests that statistical learning also contributes to creative behaviors, such as music composition (Zioga et al., 2019). Creativity is often unpredictable and uncertain because of its novelty. Thus, creativity stemming from statistical learning (hereafter, *statistical creativity*) seems to conflict with the fundamental role of statistical learning: optimizing prior prediction and suppressing uncertainty (Clark, 2013; Hasson, 2017). One possible hypothesis is that a decrease in uncertainty could act as a reward (Van de Cruys and Wagemans, 2011). However, humans cannot pursue additional potential rewards from significantly less uncertain information (Berlyne, 1970). That is, humans are curious about uncertain information for the pursuit of potential rewards (Kagan, 1972). This novelty-seeking behavior encourages the perception and production of statistically uncertain and new information, resulting in a certain degree of increase in uncertainty. People expect potential rewards from novel information with a certain degree of uncertainty and may approve of creativity. In the end, human behavior may display “fluctuation” (temporal dynamics) of uncertainty under the competition between uncertainty resolution and the further pursuit of rewards.

This article reviews neural and computational studies on the emergence of statistical creativity in the brain. In particular, we propose a hierarchical model of statistical learning: statistically chunking into a unit (hereafter, “*shallow*” statistical learning) and combining several units (hereafter, “*deep*” statistical learning). We propose a hypothesis that deep statistical learning and the fluctuation of perceptual uncertainty dominantly contribute to statistical creativity. Considering that statistical learning is fundamental to brain development, we also discuss how typical versus atypical brain development modulates hierarchical statistical learning and statistical creativity. Finally, we explore musical statistical creativity and how it interacts with general creativity (e.g., thinking and idea generation).

## From Statistical Learning to Statistical Creativity

### Prediction and Statistical Learning

The brain is a learning machine that continually adapts to varying and uncertain environments worldwide. Through learning, the developing brain gradually becomes able to comprehend and produce structured information, such as music. Predictive coding, currently a predominant theory on sensory perception (Friston, 2010; Heilbron and Chait, 2018), provides a neurophysiological architecture of predictive learning processes in the human brain. Neural representations in the higher levels of cortical hierarchies can be used to predict plausible representations in the lower levels in a top-down manner and are then compared between the hierarchies to assess the prediction error (i.e., a mismatch between a prior prediction and the actual sensory input) (Mumford, 1992; Rao and Ballard, 1999; Kiebel et al., 2008). The resulting mismatched signal is passed back up the hierarchy to update higher representations and yield better predictions. Over the long term, this recursive exchange of signals reduces the prediction error and uncertainty in the environment. In this framework, the reliability of the prior prediction is also controlled by the precision (confidence) of prediction at higher levels of a hierarchical model (Friston, 2008). This precision can be estimated by the variance of any possible sensory input, which is sometimes referred to as *perceptual uncertainty* (information entropy, Shannon, 1948). In other words, the brain perceives and suppresses the uncertainty. The expected reduction of uncertainty has generally been referred to as *salience*, evaluated from the gap between the prior and posterior distributions (i.e., Kullback–Leibler divergence or relative entropy).

Statistical learning mechanisms in the brain appear to agree with this predictive process (Harrison et al., 2006). Statistical learning is an automatic computing system by which the human brain extracts statistical regularities from the world and predicts a future state to minimize sensory reactions and uncertainty over the environment. Specifically, the brain calculates the transitional probability and precisely perceives the uncertainty of its probability distribution. This internalized probabilistic model allows us to generate prior predictions of future states and continually update the internal model (prior distribution) for better prediction and precision (Daikoku et al., 2017) by integrating sensory input with prior distribution. Evidence has also suggested that human pitch prediction of novel melodies is closely linked to statistical models of transitional probability sampled from a large corpus of music (Pearce and Wiggins, 2006, 2012; Pearce et al., 2010). This may imply that human brains acquire a statistically universal model of music through musical statistical learning.

Some researchers have suggested two interdependent processes as hallmarks of statistical learning (Rogers and McClelland, 2004; Altmann, 2017; Daikoku, 2019a,b): the chunking of statistically coherent events and the sequential combination of the chunked units. They indicated that an individual’s experience is abstracted on a statistical basis to generate a chunk that captures the statistical common and shareable denominator across individually experienced information (Sloutsky, 2010). This suggests that statistical learning underlying chunk formation and word acquisition consists of statistical accumulation across multiple episodes. However, an opposing statistical learning process appears to occur simultaneously: chunked units can be integrated to generate novel information through statistical learning (Altmann, 2017). Thus, language/music learning requires a route from the individual experience of statistical abstraction as a shareable knowledge unit (e.g., word), while comprehension and creation (e.g., grammar and sentences) require the integration of several units. Therefore, these two interdependent processes are necessary for a complete account of statistical learning and production that results therefrom (Thiessen et al., 2013; Wiggins and Sanjekdar, 2019; Wiggins, 2020).

### Statistical Creativity

Recent studies claim that statistical learning contributes to creative behaviors and learning, such as music composition (Daikoku, 2019b; Zioga et al., 2019); however, the underlying mechanisms remain unclear. In this study, we refer as creativity stemming from statistical learning as statistical creativity and propose two potential keys to statistical creativity. The first is the interplay between the chunking of statistically coherent events into a unit and the integration of several units. This process forms a hierarchical structure in statistical learning (hierarchical statistical learning). The second is the fluctuation of the perceptual uncertainty. The brain appears to seek a suboptimal solution of uncertainty for creativity based on prior predictions, which results in fluctuations in uncertainty. Furthermore, it is assumed that these two key factors interact with each other.

#### Deep Statistical Learning

Statistical learning underlying chunk formation consists of statistical accumulation across multiple episodes, contributing to generalization and abstraction (shallow statistical learning). Alternatively, an opposing statistical learning process is as follows: the integration of the chunked units could allow not only for learning of relationships between units but also the “creation” of novel information (deep statistical learning). Through statistical integration, humans can create and perceive a novel episodic representation (Altmann, 2017). We hypothesize that this deep statistical learning has a potential link to statistical creativity.

**FIGURE 1:**
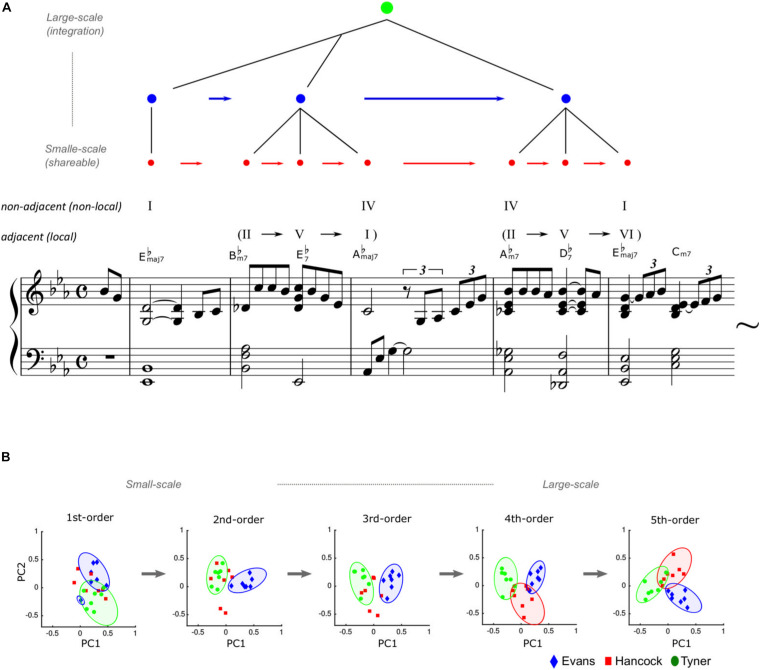
Statistical creativity in musical improvisation. Misty by Errol Garner, composed in 1954. **(A)** The arrangement, chord names, and symbols are simplified (just major/minor, *flat*, and 7th note) to account for the two-five-one (II*–*V*^(7)^–I*) progression. The statistical characteristics of jazz improvisation played by Bill Evans, Herbie Hancock, and McCoy Tyner. **(B)** Adapted from a figure of a previous article (Daikoku, 2018b). The component loading of principal component analyses showed that statistically coherent units have general characteristics shared among the three improvisors, whereas large-scale statistical units provide individualities unique to each improvisor. This suggests that abstraction (i.e., statistical learning within words) may fundamentally provide general knowledge, while integration (i.e., deep statistical learning between words) contributes to musical creativity and individuality, as well as common knowledge.

This hypothesis has been investigated in neural (Daikoku et al., 2016, 2017) and computational studies (Daikoku, 2018b, 2019b,c,d; Daikoku and Yumoto, 2020). One useful model of creativity comes from musical improvisation, in which musicians spontaneously create novel melodies and rhythms. For example, based on a computational model of the brain’s statistical learning, a study examined the statistical characteristics of jazz improvisation played by Bill Evans, Herbie Hancock, and McCoy Tyner, who are world-famous jazz pianists (Daikoku, 2018a,b). The results showed that small-scale statistical units have general characteristics shared among the three improvisers, whereas larger-scale statistical units provide individualities unique to each improviser (Figure 1B). This may suggest that small-scale (shallow) statistical learning (Figure 1A) fundamentally provides general and common knowledge, while large-scale (deep) statistical learning contributes to individual knowledge as well as common knowledge in musical creativity (Daikoku, 2019a,b). Given these findings, deep statistical learning may contribute mainly to individual phrasing or melody, while small-scale statistical learning may underlie the production of several tone transitions and consistent rhythm properties.

For example, jazz music has general regularities in chord sequences such as the so-called “two-five-one (II–V–I) progression.” This is a common cadential chord sequence used in a wide variety of music genres, including jazz harmony. It is a succession of chords whose roots descend in fifths from the supertonic (II) to dominant (V), and finally to the tonic (I). Such syntactic progression frequently occurs in a jazz improvisation, and therefore, the statistics of the sequential information have high transitional probability and low uncertainty. Thus, once a person has learned the statistical characteristics, it can be chunked as a commonly used unit among improvisers. In contrast, the ways of combining the chunked units are different between improvisers and therefore represent the individuality of musical creativity (see Figure 1).

In this phrase of Figure 1A, the chord “IV” (E♭maj7) in the fourth measure corresponds to the chord “I” (E♭maj7) in the second measure occurring several chords earlier, creating a non-adjacent hierarchical dependency between “I” and “IV” in a recursive fashion. The local dependency between the first and second chords (E♭maj7 – B♭m7) is less likely according to traditional music theory, but this second chord lays the groundwork for the non-local dependency between “I” and “IV” by generating a II–V–I progression (i.e., B♭m7 – E♭7 – A♭maj7). Another type of interaction can be seen in the latter half of the phrase (i.e., adjacent: II – V – VI – IV, non-adjacent: IV –I). Near the end of the piece, the higher hierarchy of the harmony structure “I – IV (– IV) – I” nests the lower hierarchy of the structures “II–V–I” and “II–V–VI–IV.” Hofstadter (1979) also indicated that a key change embedded in a superordinate key forms hierarchical non-adjacent structures in a recursive fashion. Thus, composers generally design hierarchical non-adjacent structures in a recursive fashion, potentially using this technique to organize the entire movement of a symphony or sonata (Schenker and Jonas, 1956).

To summarize, hierarchical statistical learning is as follows: The interplay between the chunking of statistically coherent events and the integration of several units could form hierarchically structured information, such as music. Hierarchical statistical learning is a window of these deeper processes that underpin creativity (Altmann, 2017). It is assumed that deep and large-scale statistical learning may contribute significantly to statistical creativity (Table 1). However, it is noteworthy that the individuality of musical representations does not necessarily contribute to musical creativity. Creativity is the process of producing new and worthwhile information. In this concept, a fixed representation of individual knowledge can also be interpreted as less creative and less uncertain. The flexibility of the presentation is crucial for producing novel and uncertain information. To discuss how the representation of individual knowledge that emerges from deep statistical learning interacts with their musical creativity, the next section proposes the second key to statistical learning: temporal dynamics of perceptual uncertainty.

**TABLE 1:** Summary of our two proposed levels of statistical learning and statistical creativity.

	**Deep**	**Flat (shallow)**
Learning	Syntactic and integration of chunked units	Lexical, chunking, and abstraction
Memory	Large-scale and individual	Small-scale and shareable
Production	Creativity	Generality and commonality
Development	Typical ≠ atypical	Typical ≃ atypical

#### Temporal Dynamics of Perceptual Uncertainty

Another key insight into statistical creativity is the fluctuation (temporal dynamics) of perceptual uncertainty. Perceptual uncertainty can generally be estimated by the variance of any possible sensory input (i.e., prior distribution; see section “Prediction and Statistical Learning”). The brain is motivated to optimize prior predictions and minimize uncertainty by learning (Friston, 2010). The decrease in uncertainty generally delivers pleasure, acting as a reward (Van de Cruys and Wagemans, 2011). In other words, humans are curious about uncertain information about potential rewards (Kagan, 1972). We hypothesize that such novelty-seeking behavior motivates the perception and production of novel and uncertain information. People are expected to receive potential rewards from novel and uncertain information and may approve such information as creativity. Through this competition between uncertainty resolution and the pursuit of rewards, human behavior may display fluctuations in uncertainty. Furthermore, perceptual uncertainty is based on sensory input, but it can also be an internal input. That is, the internal mental imagination of a new idea may also occur without sensory input, relying only on the uncertainty of the internalized statistical model.

**FIGURE 2:**
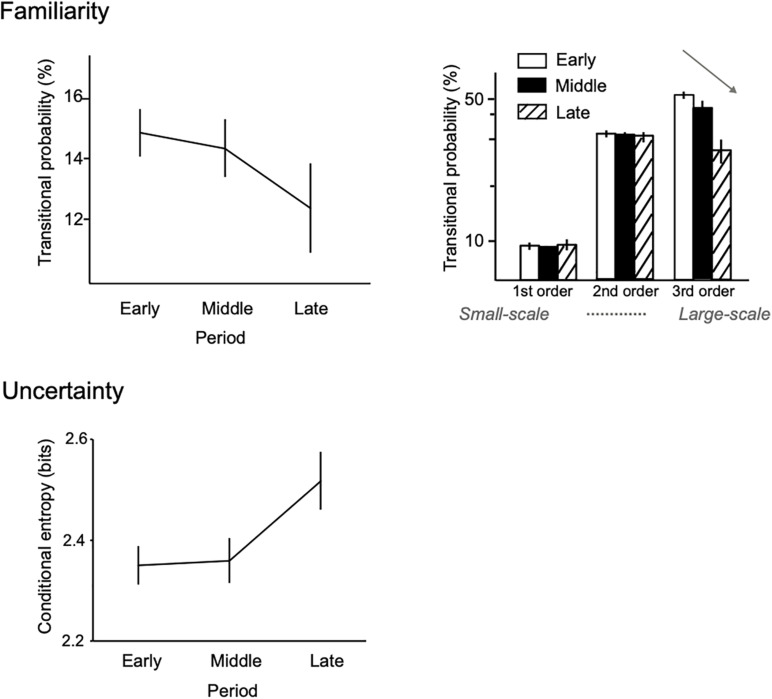
Statistical creativity in the uncertainty fluctuation of musical composition. Figure adapted from a previous article (Daikoku, 2019b). From the early to the late periods of Beethoven’s lifetime, the predictable patterns that ubiquitously appear in all of his piano sonatas (familiar sequence) were decreased, whereas the uncertainties were gradually increased. Further, these findings were more prominent in higher- (deeper), rather than lower-order statistical learning models (right). This may suggest that higher-order statistical learning reflects novelty-seeking (creative) behavior over a composer’s lifetime.

Recent theories (Huron, 2006) and studies (Egermann et al., 2013; Koelsch, 2014; Gingras et al., 2016) suggest that the temporal dynamics of uncertainty may contribute to the esthetic appreciation of art and music and that this fluctuation may encourage humans to create and learn new regularities (Schmidhuber, 2006). For example, computational evidence shows that the uncertainty of music (conditional entropy of music sequence) fluctuates over a composer’s lifetime (Daikoku, 2018d, 2019b). In these studies, across Beethoven’s lifetime, the frequency of predictable patterns that are ubiquitous in his piano sonatas (familiar phrases) was found to decrease, whereas the entropy of statistical distribution gradually increased (Figure 2). Furthermore, these findings were more prominent in large-scale and deep statistical learning (see section “Deep Statistical Learning” and Table 1). This suggests that deep statistical learning is sensitive to the emergence of creativity as well as individuality. These findings may be explained from the viewpoint of the Wundt curve, as described by Berlyne (1970). This suggests that the hedonic value of complex stimuli tends to rise as they become less novel, while the opposite holds true for simple stimuli. This means that if familiarization of stimuli had proceeded further, the interestingness of the simple patterns would have continued to decline, whereas those of the complex patterns would have climbed to the peak of a Wundt curve. To summarize, creative behavior does not necessarily generate information—theoretically optimal, efficient, and certain information; instead, it sometimes gives rise to uncertain and unpredictable information.

### What Is Musical Creativity?

**FIGURE 3:**
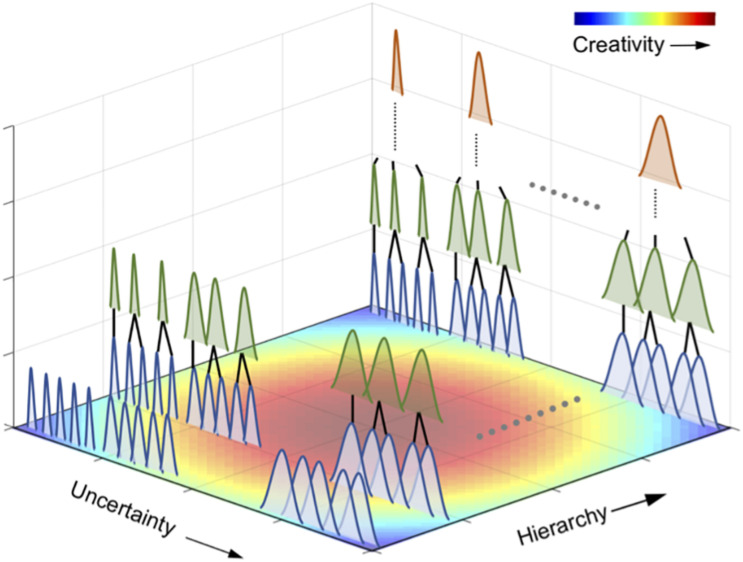
A hypothesis of statistical creativity. Statistical creativity may, at least, be achieved via two potential mechanisms in a hierarchical statistical learning. The first is the interplay between the chunking of statistically coherent events into a unit and integration of the several units. This process forms a hierarchical structure in statistical learning (i.e., hierarchical statistical learning). The second is a perceptual uncertainty as shown in each of the bell-shaped distribution in the figure. The brain appears to seek a suboptimal solution of uncertainty for creativity based on prior distribution in the internal predictive model. It is assumed that a perceptual uncertainty at not very small- and large-scale statistical learning may induce statistical creativity.

We emphasize that statistical learning plays a key role in musical creativity. In particular, we propose two important roles for statistical learning in musical creativity. The first is a hierarchy of shallow and deep statistical learning. As discussed, small-scale (shallow) statistical learning (Figure 1A) may fundamentally provide general and common knowledge, while large-scale (deep) statistical learning contributes to individual knowledge of music (Daikoku, 2019a,b). In general, deep statistical learning is a mechanism for the integration of chunked units acquired by shallow statistical learning. That is, deep statistical learning of music could occur after persons have robust shallow statistical models of chunks. From the information theoretical perspective, as the order of transitional probability in the Markov chain becomes higher (i.e., the scale is larger), transition patterns can also be subdivided (for more detail, see Figure 3B of Daikoku, 2018c). That is, there are more sequential patterns in the deeper model. This leads to a diversity of patterns and individuality in music and possibly leads to musical creativity. Thus, deep statistical learning (integration of chunked units) may allow for the creation of a novel melody and rhythm even in the absence of any prior knowledge.

The second is a fluctuation in uncertainty. In general, creativity is defined as a process of producing something that is both original and worthwhile (Lubart and Mouchiroud, 2003; Kozbelt et al., 2010; Robert, 2011). Due to its novelty, creative information is often unpredictable and uncertain. It has been suggested that novel and uncertain musical information emerges through hierarchical statistical learning. However, there is still little understanding as to why such uncertain information can be accepted as creative. In other words, highly uncertain information is not necessarily creative. For example, a random tone sequence is highly uncertain, but in general, we do not approve of a random time sequence as creative music. Hence, it is assumed that appreciation of musical creativity may be associated with certain forms of suboptimality between uncertainty and certainty (Figure 3). We hypothesize that such competitive pursuits of uncertainty and certainty may induce fluctuations in uncertainty and that fluctuations in uncertainty may contribute to musical creativity.

Evidence has revealed that musicians are good statistical learners (Francois and Schön, 2011; Paraskevopoulos et al., 2012; Elmer and Lutz, 2018; Daikoku and Yumoto, 2020), allowing the brain to precisely grasp the temporal dynamics of uncertainty in music perception and production (Hansen and Pearce, 2014; Daikoku, 2019b; Zioga et al., 2019). We hypothesize that such proficiency in precision in perceptual uncertainty may also allow musicians to control the uncertainties in music finely by manipulating several musical components such as rhythm, melody, and harmony. Musical tensions can be created by establishing a predictable pattern in rhythm and melody and subsequently denying the prediction from it (Meyer, 2008). We can derive pleasure from deviant and uncertain musical patterns once a predictive pattern is established. Evidence suggests that so-called “music chills” are correlated with violations of expectation (Sloboda, 1991) and underpin musical appreciation (Huron, 2006). A neural study revealed that music chills increase brain activity in reward areas (ventral striatum) and decrease activity in the amygdala and ventromedial prefrontal cortex (Blood and Zatorre, 2001). This suggests that we derive rewards from violations of expectations, as well as from confirmed predictions. It is suggested that such esthetic appreciation can be reflected in the temporal dynamics of uncertainty.

Alternatively, musicians who have trained for a long period may have robust internal statistical models of music (Hansen and Pearce, 2014). Furthermore, a study has suggested that the characteristics of internal models respond to one’s own musical culture, such as Japanese and Western classical music (Daikoku et al., 2020). This may lead to cultural fixation of statistical knowledge and even bolster productivity instead of creativity. Statistical learning has been shown to be ubiquitously performed regardless of the intention (Perruchet and Pacton, 2006; Tsogli et al., 2019). This suggests that statistical knowledge is influenced by surrounding environmental information. Nevertheless, such musicians aptly exhibit pathways of high creativity (Kleinmintz, 2017; Przysinda et al., 2017; Zioga et al., 2019). One possible reason is that the knowledge and behavior that results from statistical learning involve implicit mechanisms with less intention (Perruchet and Pacton, 2006; Paraskevopoulos et al., 2012; Koelsch et al., 2016; Christiansen, 2019) but can transform into explicit knowledge through long-term training and experience (Batterink et al., 2015; Moser et al., 2020). Statistical learning of behavior is also considered as procedural learning that takes place without explicit knowledge (Kóbor et al., 2018). Therefore, we hypothesize that musical creativity resulting from statistical learning is mainly involved in intuitive performance, such as musical improvisation, in which musicians intuitively play new melodies and rhythms (Daikoku, 2018b).

Musical creativity is likely to be correlated with general creativity. A previous study examined how jazz improvisers, non-improvising musicians, and non-musicians perform the domain-general task of divergent thinking as well as the musical task of preference ratings for chord progressions that vary in expectation (Przysinda et al., 2017). The results showed that jazz musicians preferred unexpected (unpredicted) chord progressions. Further, the unexpected stimuli elicited larger music expectancy-related neural responses (early right-anterior negativity: ERAN) and another event-related potential (ERP) of P3b, followed by smaller long-latency responses (late positivity potential) in jazz musicians. This implies that people who can predict precisely a musical event prefer an unpredictable one, possibly because they can correctly discriminate between familiar and novel musical events (i.e., creative). Notably, these neural effects were significantly correlated with fluency and originality in the divergent thinking task. This suggests that the precision of (prior) prediction is crucial for general and musical creativity.

## Neural Perspectives of Statistical Creativity

Recently, an increasing number of studies have suggested neural mechanisms of creativity. In particular, they showed that prefrontal function and some types of neural networks are associated with human creativity. In this section, by reviewing a number of neural studies, we discuss how the frontal functions and the three types of neuronal networks contribute to statistical learning and statistical creativity.

### A Role of Frontal Cortex in Prior Prediction and Creativity

Frontal lobe functions are considered to be one of the most important keys to understanding creativity in the brain (Flaherty, 2005) and is generally involved in the top-down control of executive functions and decision-making (Gold and Shadlen, 2007; Dosenbach et al., 2008; Heekeren et al., 2008; Dalley et al., 2011). Recent studies have suggested that the prefrontal lobe (e.g., the inferior frontal gyrus, IFG) and dorsal connectivity between the prefrontal and sensory areas are associated with the formation of internal Bayesian models and prior predictions (Friston et al., 2016; Cope et al., 2017; Park et al., 2018). According to their studies, Bayesian models (i.e., prior prediction) could be generated in IFG and/or frontal motor speech regions and conveyed to auditory sensory regions through synaptic connections to instantiate plausible representations.

This hypothesis may also be explained by the developmental processes. A recent study indicated that this prefrontal-auditory connectivity is better developed in human adults than in newborns and macaques (Friederici et al., 2017). They also showed that in newborns, only the dorsal stream terminates in the premotor cortex (PMC). This partially supports the computational hypothesis that infants may have a prior prediction. That is, the development of the brain allows us to switch from a strong reliance on sensory input and weak reliance on prior predictions (hypo-prior) at an early learning stage to proper integration of sensory information with prior prediction (internal model) at later learning stages, becoming robust against disturbances in the uncertain phenomena (Philippsen and Nagai, 2019). Infants may have hypo-prior prediction due to the prematurity of dorsal prefrontal-sensory connectivity, which is essential for generating prior prediction and integrating prior prediction with sensory input. Together, many pieces of evidence suggest that prefrontal function may contribute to strong dependence on top-down prior prediction in perceiving and producing information. Such predictions can be generated by the acquired knowledge and experience. Hence, the strong dependence on prior prediction is partially interpreted as a strong reliance on certain acquired knowledge. Neural evidence has shown that both large- (deep) and small-scale (shallow) statistical learning involve top-down prior prediction (Daikoku et al., 2017). The magnetoencephalographic (MEG) study suggested that both mechanisms combine statistically chunks into a unit (small-scale statistical learning) and several units (large-scale statistical learning) that are reflected in mismatch responses.

However, prior predictions may sometimes inhibit creativity. Creativity is a phenomenon whereby something new and uncertain is formed, even if creativity is intricately linked to acquired knowledge. Therefore, the inhibition of prefrontal function may partially induce creative and uncertain information production (Chrysikou, 2018), possibly because of less dependence on prior prediction and certain knowledge. The neural evidence seems to agree with this hypothesis. Electroencephalography (EEG) (Fink et al., 2006, 2009; Lustenberger et al., 2015) and functional magnetic resonance imaging (fMRI) studies (Bengtsson et al., 2007; Berkowitz and Ansari, 2008; Limb and Braun, 2008; de Manzano and Ullén, 2012a,b) have examined brain activity during exposure to fixed melodies (less creative) or free-improvised melodies (more creative). The results indicate that more creative conditions lead to stronger alpha power (Fink et al., 2006; Lustenberger et al., 2015; Lopata et al., 2017) in the right frontal and parietal regions (Fink et al., 2009). The increased oscillatory activity in the alpha band is considered to reflect inhibition of the top-down process (Klimesch, 2012). However, other studies have suggested that alpha power reflects internally oriented attention, in which external bottom-up stimulation is also suppressed (Fink and Benedek, 2014). One study that investigated both the neural and genetic correlates of creativity suggested that a system of interaction between strong top-down and weak bottom-up processes underpins creativity, which is modulated by competition between the glutamate and GABA neurotransmitter systems (Liu et al., 2018). Furthermore, a computational model (Collins and Koechlin, 2012) inspired the hypothesis that the frontal lobes create an expanding repertoire of flexible behavioral strategies for driving action in uncertain, changing, and open-ended environments and suggested that frontal lobe function, including executive control and decision-making, somewhat supports the integration of reasoning, learning, and creativity through uncertainty monitoring. Green et al. (2017) also suggested that neural activity in the frontopolar cortex facilitates creative intelligence.

The contradiction between these two opposing findings on inhibition and enhancement of top-down control may be explained by the different tasks set in the different studies (Adhikari et al., 2016). In fMRI studies (Pinho et al., 2015), improvisation using a defined pitch set resulted in activation of the dorsolateral prefrontal cortex (dlPFC) because participants had to maintain available note choices in their working memory. In contrast, free improvisation leads to deactivation of the dlPFC because participants are able to take advantage of their implicit learning systems to create improvisations in which top-down control from the dlPFC would be disadvantageous (Dhakal et al., 2019). Using fMRI, Liu et al. (2015) examined brain mechanisms during poetry composition and the assessment (revision) process. The results indicated that dlPFC activity was attenuated during composition and reengaged during revision, whereas the medial prefrontal cortex (MPFC), which is associated with multiple cognitive functions such as motivation (Kouneiher et al., 2009) and unconscious decision-making (Soon et al., 2008), was active during both phases. Furthermore, expert poets showed significantly stronger deactivation of the dlPFC during composition, but there was no significant difference in the activity of the MPFC. Thus, expert poets may more effectively suspend top-down control while maintaining their motivation. Together, these findings show that open-ended creative and uncertain behaviors may suppress top-down controls, as expressed through the dlPFC activity level, while maintaining motivation, as expressed through MPFC activity level, whereas fixed behaviors enhance top-down control.

### A Role of Neural Network in Temporal Dynamics of Perceptual Uncertainty and Creativity

Evidence suggests that the temporal dynamics of creativity processes are reflected in three types of neuronal networks (Beaty et al., 2018). First, the default mode network (DMN), which consists of the cortical midline and posterior inferior parietal regions, underpins spontaneous idea generation, episodic future thinking, and mind-wandering, among others (Mason et al., 2007; Zabelina and Andrews-Hanna, 2016). Second, the executive control network (ECN), which involves the lateral prefrontal and anterior inferior parietal regions, contributes to idea evaluation and executive function (Beaty et al., 2016). Third, the salience network (SN), which consists of the bilateral insula and anterior cingulate cortex, plays a role in conveying candidate ideas originating from the DMN to the ECN for idea evaluation (Beaty et al., 2016, 2018).

A previous study demonstrated that creative people show higher global efficiency within these networks, that is, a smaller number of paths traverse between brain regions (Beaty et al., 2015). In other words, the efficiency of the interplay between idea generation and evaluation is higher in creative people (Kleinmintz et al., 2019). Importantly, the perceptions of novelty (and surprise) are involved in both idea generation and evaluation processes, but not either of them; when generating a new idea, they need to recognize that it is a novel idea, not to mention when evaluating. This previous finding may explain the contradiction between inhibition and enhancement of frontal activity during creative behavior, as discussed in section A Role of Frontal Cortex in Prior Prediction and Creativity.” Creative people have the ability to simultaneously engage these large-scale brain networks, including the DMN, ECN, and SN (Boccia et al., 2015; Beaty et al., 2018). It is assumed that creativity is not just free and uncontrolled activities but rather elaborate collaboration between uncontrolled/uncertain mind activity (i.e., DMN), which is less dependent on frontal function, and the top-down executive control of free thinking, including frontal function (i.e., ECN).

Together, the prefrontal function and three types of neural networks may have an important role in statistical creativity, particularly in terms of perceptual uncertainty. We hypothesize that the inhibition of prefrontal function may induce creative and uncertain information production, possibly because of the weakened dependence of prior knowledge. Besides, it is assumed that sophisticated creativity is not just free-thinking activities uncontrolled by prior knowledge but rather an elaborate collaboration between uncontrolled/uncertain mind activity (i.e., DMN), which is less dependent on frontal function, and top-down executive control of free thinking, including frontal function (i.e., ECN).

## Statistical Creativity in Atypical Development

Statistical learning is essential for brain development, as infants can implicitly perform statistical learning to acquire their native language (Teinonen et al., 2009). Computational studies allow modeling of the brain’s developmental processes in predictive functions. Evidence suggests that the development of the brain allows us to switch from a strong reliance on the statistics of sensory input along with weak reliance on prior predictions (hypo-prior) to a proper integration of sensory statistics with prior prediction (internal model), thus becoming robust against disturbances in an uncertain environment (Philippsen and Nagai, 2019).

However, developmental disabilities, such as autism spectrum disorder (ASD), may develop different neural mechanisms underlying prior prediction (Nagai, 2019; Lanillos et al., 2020). For example, some studies have suggested that individuals with ASD have hyper-plasticity in short-term statistical learning, such that they prefer recent sensory statistics rather than global (i.e., long term) statistical structures in sequential information (Sinha et al., 2014; Saffran, 2018). Thus, individuals with ASD are likely to show a strong reliance on sensory input and weak reliance on prior prediction (i.e., hypo-prior or hypersensitivity) in statistical learning. Notably, there is likely a contrastive type of abnormal development of predictive function: a stronger reliance on prior predictions (i.e., hyper-prior) (Philippsen and Nagai, 2019) than hypo-prior predictions (Pellicano and Burr, 2012). That is, the abnormality of prior prediction in ASD can be characterized by instability or variability, rather than either enhancement or decay, of reliance on prior prediction as compared to typical development (TD).

Such instability of reliance on prior prediction could also influence the precision of perceptual uncertainty because the precision is estimated by the variance of any sensory input (i.e., prior distribution). Some studies have indicated that ASD is susceptible to perceptual uncertainty (Boulter et al., 2014; Lawson et al., 2014; Van de Cruys et al., 2014). Uncertainty intolerance can be postulated as a key marker of generalized anxiety disorder (Freeston et al., 1994). The strong anxiety, observed as a common property of ASD, may also be explained by the intolerance of uncertainty and influence creativity (Baas et al., 2008). One study claims that such anxiety in ASD should emerge when environmental uncertainty is high (Boulter et al., 2014).

Thus, atypical brain development may exhibit specific characteristics (rather than decay or facilitation) of their statistical learning abilities. It is assumed that such specificity of statistical learning abilities could affect statistical creativity as well as prior prediction and perceptual uncertainty. A number of studies have reported that people with ASD sometimes exhibit superiority in some abilities (Boucher et al., 2012), such as mathematics, visual search skills (O’Riordan et al., 2001), and music and art skills (Happé and Frith, 2009; James, 2010). Furthermore, the right hemispheric networks are strongly dominant in ASD (Mason et al., 2008) and musicians (Zatorre et al., 2002). It has been thought that the right hemisphere function plays an important role in musical performance. It is possible that the dominance of the right hemisphere in individuals with ASD may influence their capacity for musical creativity.

A previous study showed that individuals with ASD can think of more unusual, uncertain ideas in divergent thinking tasks, although they produce fewer ideas than TD people (Best et al., 2015). Neural evidence may partially support this finding: the brain in ASD has hypoconnectivity between the prefrontal cortex and other areas (Belmonte et al., 2004; Just et al., 2004; Courchesne and Pierce, 2005; Green et al., 2020). Prior prediction mainly originates in frontal regions and is transmitted to sensory regions through synaptic connections (Cope et al., 2017; Park et al., 2018). The connectivity between the frontal and sensory areas is considered to play an essential role in conveying prior predictions to instantiate a plausible representation of sensory input. The brains of individuals with ASD may alter this connection (Belmonte et al., 2004; Just et al., 2004; Courchesne and Pierce, 2005; Green et al., 2020). This alteration leads to the modulation of the prior prediction. Nevertheless, the inhibition of prefrontal function may induce uncertain information production, possibly due to the modulation or depletion of prior prediction (hypo-prior).

Another key insight is deep and large-scale statistical learning (integration of chunked units). Evidence suggests that people with ASD display abnormalities in episodic memory representations (Goh and Peterson, 2012). Episodic representations are generally large-scale compared to semantic representations, such as words. A neuroimaging study also showed that the DMN, which is an important network for creativity, is altered in the brain in ASD; further, this alteration can lead to atypical integration of information about the self in relation to others (Padmanabhan et al., 2017). Furthermore, individuals with ASD may show inconsistent MMN responses to local (i.e., small-scale) deviants; some studies found weaker MMN in ASD than TD (Seri et al., 1999; Abdeltawwab and Baz, 2014; Bonnet-Brilhault et al., 2016), while other studies detected larger MMN in ASD than in TD (Gomot et al., 2002, 2011; Ferri et al., 2003; Lepistö et al., 2005; Green et al., 2020). Given these findings, individuals with ASD have either hyposensitivity or hypersensitivity to local sensory properties. In contrast, individuals with ASD seem to show consistent findings on global (i.e., large-scale) predictive processing: a study indicated weak MMN responses to global deviants (Goris et al., 2018). This may imply that ASD is hyposensitive to larger-scale statistical learning, while sensitivity to local events depends on the type of stimuli (Ide et al., 2017), representing either hypo/hypersensitivity to small-scale and local statistical learning.

In summary, atypical alterations in prior prediction and perceptual uncertainty may lead to individual characteristics of statistical creativity. Further research focused on the individuality of creativity that may illuminate the potential otherness of creative ability.

## Discussion

In this study, we propose a hierarchical model of statistical learning: statistically chunking into a unit (shallow statistical learning) and combining several units (deep statistical learning). We hypothesized that (Table 1):

(a) Large-scale statistical learning contributing to individual deep knowledge.
(b) Temporal dynamics of uncertainty, representing a suboptimal solution for creativity.

can be a potential causal factor in statistical creativity. Figure 3 presents an overview of the hypotheses in this study. It is proposed that perceptual uncertainty at not exceedingly small- and large-scale statistical learning may induce statistical creativity. Statistical creativity may, at least, be achieved via two potential mechanisms. The first is the integration of the chunked units, which could allow not only for learning of relationships between units but also the “creation” of novel information (“*deep*” statistical learning). That is, we can generate new information (e.g., sentences) by integrating common knowledge (e.g., words). This process also allows for a hierarchical structure in statistical learning. The second is the temporal dynamics (fluctuation) of perceptual uncertainty, as shown in each bell-shaped distribution in Figure 3. The brain appears to seek a suboptimal solution of uncertainty for creativity based on prior distribution. We also hypothesize that the first and second mechanisms of statistical creativity interact with each other. That is, the fluctuation of uncertainty may arise through the interplay between shallow and deep statistical learning, resulting in increased uncertainty.

It is also noteworthy that the two factors of statistical creativity are potentially correlated with neural bases. The prefrontal function and three types of neural networks may play an important role in statistical creativity, particularly in terms of perceptual uncertainty. The suppression of prefrontal function may induce creative and uncertain information production, possibly because of the weakened dependence on prior knowledge. However, elaborated creativity is not just free and uncertain thinking with less contribution from prior knowledge, but rather a collaboration between free thinking and certain prior knowledge. It is assumed that such collaboration is partially reflected in the temporal dynamics of uncertainty in a certain degree of deep statistical creativity (Figure 3).

Statistical learning is thought to be a domain-general and species-general learning principle that occurs for visual and auditory information, including language and music, and in both primates and non-primates, such as songbirds (Lu and Vicario, 2014, 2017), monkeys (Saffran et al., 2008), and rats (Toro et al., 2005). The current statistical learning hypothesis, however, may not be sufficient to cover all levels of music processing, including domain-specific mechanisms such as universal grammar, tonal pitch spaces, and hierarchical tension (Hauser et al., 2002; Jackendoff and Lerdahl, 2006). Some studies suggest that there are two steps in the learning process (Jusczyk, 1999; Ellis, 2009). The first is statistical learning, which shares a common mechanism among all domains (domain generality). The second is domain-specific learning, which has different mechanisms in each domain (domain specificity). Nevertheless, it is still unknown how statistical learning interacts with domain-specific learning, how various aspects of statistical learning (i.e., abstraction of statistically coherent events vs. combining the chunked units and shallow and deep levels) are linked to top-down and bottom-up processes of the brain, and how statistical knowledge can be used in creativity. Further, although creativity is associated with perception as well as production (Dailey et al., 1997; Furlong, 2009), no study has fully revealed the precise distinctions between creative production and perception (Hargreaves, 2012) from a statistical learning framework.

Categorization (Jones and Mewhort, 2007) and non-adjacent dependency (Frost and Monaghan, 2016) are likely to be the key mechanisms for understanding these questions. For example, humans learn the transitional probabilities of word categories, such as nouns and verbs (Jones and Mewhort, 2007); when the verb “drink” occurs, the brain predicts many subsequent words which can be drunk. The brain can also generalize both adjacent and non-adjacent statistical rules of grammar and apply these rules to novel vocabulary (Gomez and Gerken, 1999). Using such mechanisms, the brain does not have to code all the received information, contributing to memory capacity and uncertainty reduction. We hypothesize that this information efficiency encourages humans to produce uncertain and creative information. Future studies are necessary to demonstrate the roles of hierarchical statistical learning in categorization and non-adjacent dependency.

Notably, the current statistical creativity model does not fully explain all the components necessary to be accepted as creativity. Creativity is the process of producing something worthwhile as well as original (Lubart and Mouchiroud, 2003; Kozbelt et al., 2010; Robert, 2011). Despite the evidence on the contribution of statistical learning to the production of new and uncertain information, little is understood about how and why people can recognize such information as worthwhile and creative. A recent neural study demonstrated that uncertainty and surprise jointly predict musical pleasure reflected in the amygdala and hippocampus (Cheung et al., 2019). This study suggested that musical chord with high uncertainty but low surprise, and vice versa, evoked high pleasure. Given the previous findings, we hypothesize that not remarkably high and low uncertainty can be recognized as creative and valuable information. This fundamental question will be key to understanding why people can recognize uncertain information as worthwhile and novel.

Hierarchical statistical learning may be a key insight into examining the influence of dispositional, maturational, and developmental factors of the individuality of creative ability in the brain with developmental disorders such as ASD. Statistical learning is an innate mechanism that is facilitated by postnatal musical training (Francois and Schön, 2011; François et al., 2012; Paraskevopoulos et al., 2012; Daikoku et al., 2020). There is inconsistent evidence suggesting the enhancement and reduction of statistical learning ability in brains with ASD (Gomot et al., 2011; Roser et al., 2015; Goris et al., 2018; Green et al., 2020), which is generally thought to be associated with a combination of genetic and environmental factors (Chaste and Leboyer, 2012). A previous study proposed a neurocognitive model of competence development (Seither-Preisler et al., 2014), which describes the interaction between dispositional factors, natural maturation, and training-induced neural plasticity. The authors claimed that in the case of music processing, the morphology of the auditory cortex (bottom right) and the source waveforms of the early ERP component (P1) represent dispositional and training-induced factors, respectively. A neural network that is important for creativity (i.e., DMN) has also been considered to be associated with both genetic (Meda et al., 2014) and training factors (Taylor et al., 2013). Thus, dispositional, maturational, and learning-induced factors may play a key role in the development and emergence of statistical creativity. Future research is needed to investigate how prior dispositions interact with the influence of postnatal training. We believe that this review will shed light on the key roles of statistical learning in musical creativity and facilitate further investigation on how the development of the brain modulates creativity.

## Concluding Remarks

Musical creativity is ubiquitous and unique to humans. The interaction between musical creativity and the brain is complex and involves a variety of neural circuits underlying sensory perception, learning, memory, action, and creativity. We emphasize that musical creativity engages “hierarchical” statistical learning. In particular, we propose two components that give rise to creativity. The first is deep statistical learning (integration of shareable units). The second is the temporal dynamics (fluctuation) of perceptual uncertainty. Considering evidence that the brains of individuals with ASD are susceptible to uncertainty, we assert that creativity in ASD can covertly reflect more (internally oriented) emotional representations against uncertainty and generation of creative and individual episodic information. Further research focused on the hierarchy of statistical learning and temporal dynamics of perceptual uncertainty may provide new insights into musical and general creativity in atypical and typical brains.

## Author Contributions

TD prepared the figures and wrote the original draft of the manuscript. GW and YN reviewed and edited the manuscript. TD, GW, and YN wrote the final manuscript. All authors contributed to the article and approved the submitted version.

## Conflict of Interest

The authors declare that the research was conducted in the absence of any commercial or financial relationships that could be construed as a potential conflict of interest.
